# Joseph E Murray, MD: redefining surgical success through the evolution of transplantation

**DOI:** 10.3389/frtra.2026.1995998

**Published:** 2026-10-26

**Authors:** Pia F. Koch, Stefan G. Tullius

**Affiliations:** 1 Division of Transplant Surgery, Department of Surgery, Brigham and Women’s Hospital, Harvard Medical School, Boston, MA, United States; 2 Department of Surgery, Campus Charité Mitte | Campus Virchow-Klinikum, Charité - Universitätsmedizin Berlin, Berlin, Germany

**Keywords:** Joseph E. Murray, long-term-survival, quality of life, surgery, transplantation

## Introduction

The definition of surgical success has changed profoundly over the past century. For much of surgical history, success was measured by the technical accomplishment of the operation and its immediate outcome: whether the patient survived the procedure, whether the operation could be completed safely, and whether major postoperative complications could be avoided.

Today, surgical success extends far beyond the surgical theatre: it encompasses long-term survival, durable disease control, functional recovery, and quality of life, shifting attention from the surgery itself to the life that follows.

Few developments illustrate this evolution more clearly than organ transplantation. More than a technical achievement, transplantation raised questions that began only after the surgery was completed: could a transplanted organ function for years? Could recipients return to normal life? Could a single operation become a durable therapy? Answering these questions transformed transplantation from an isolated surgical procedure into a model of lifelong care.

Dr. Joseph E. Murray played a key role in this transition. Widely remembered as the pioneer of organ transplantation, the surgeon who performed the first successful human kidney transplant in 1954, and the recipient of the 1990 Nobel Prize in Physiology or Medicine (1), Murray's contributions go beyond the advancements of organ transplantation, introducing a concept that re-defined surgical success.

## Success meant lasting benefit

The first successful human organ transplantation marked a turning point for the field of surgery.

In 1954, Richard Herrick was dying of end-stage renal disease when his twin brother Ronald offered gave him one of his kidneys (2). Their genetic identity provided a unique opportunity to overcome the immunological barrier that had defeated previous attempts. On December 23, Joseph Murray and his team performed the first clinical transplantation, successfully replacing a failing organ (2) (Figure 1). What made the achievement remarkable was not only the technical success but the broad potential it carried forward.

**Figure 1:**
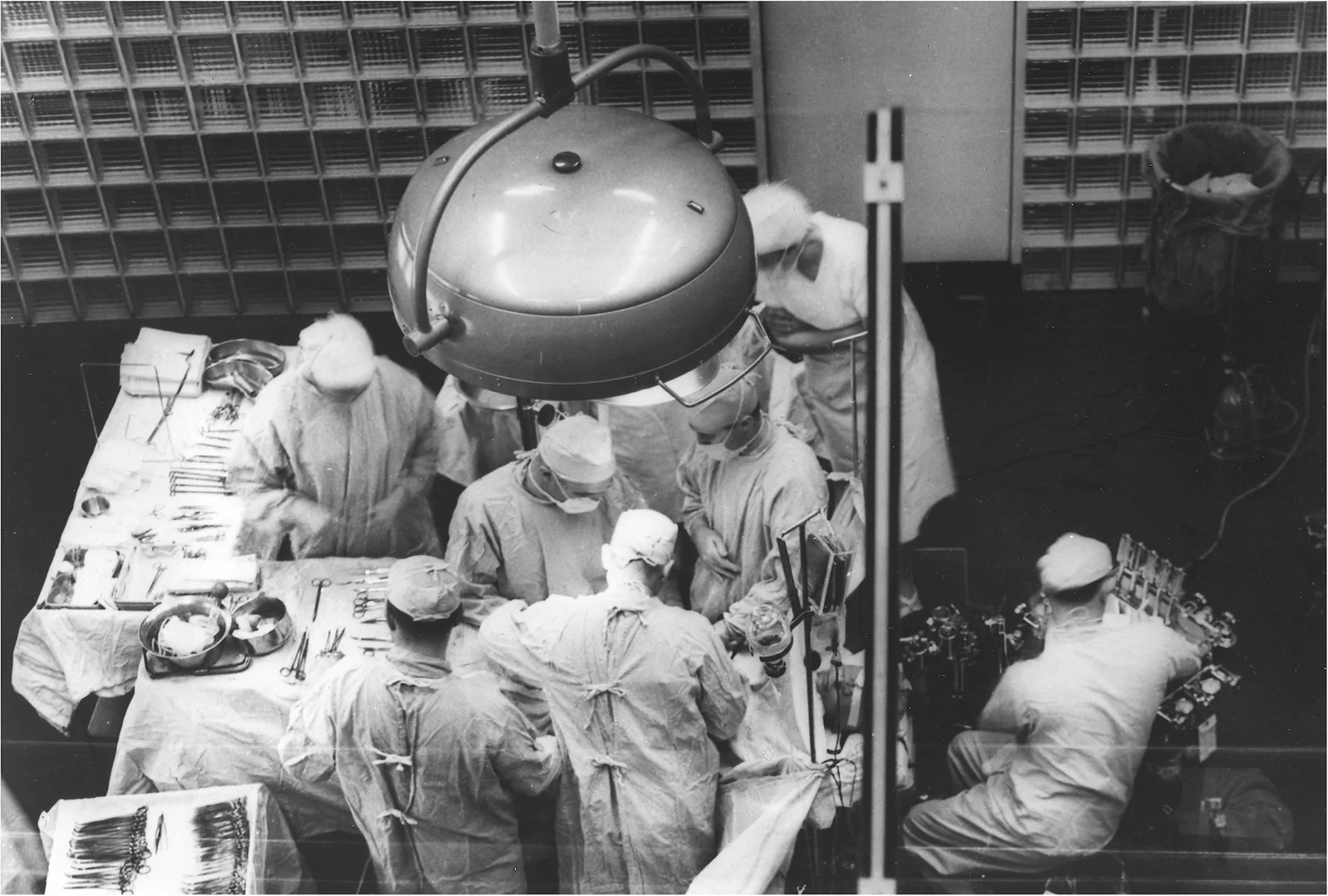
Joseph E. Murray (third from left), and colleagues during the first successful kidney transplantation at the Peter Bent Brigham Hospital, Boston, 1954. © Brigham and Women's Hospital. Reproduced with permission. Source: Brigham and Women's Hospital Archives. https://www.dropbox.com/scl/fo/popm1nugl0dr8du97ebz6/AH5LI5-0xSZlYghV0h2iWG8?rlkey=fksg16qow31vw3vy3k1assso0&dl=0 This image is not covered by the Creative Commons license applying to this article.

By the mid-twentieth century, surgery had already significantly advanced, with major progress in vascular, thoracic, and neurosurgical procedures (3). Yet, surgical achievements were still largely judged by what could be accomplished safely in the operating room. The Herrick case transformed transplantation from an experimental concept into a viable surgical therapy. Richard Herrick survived for eight years after the procedure, demonstrating that the success of surgery could be measured not only by what was accomplished during an operation, but by the life that operation made possible afterward. Indeed, Richard used his eight years effectively, married his recovery room nurse and together they had two children (4).

Yet, this exceptional success also exposed a fundamental limitation of transplantation: even the most technically successful operation could fail if the pathophysiology of the underlying disease was not understood or the post-operative treatment was not meeting the specific biological needs.

## Success meant understanding the immunobiological consequences

This next challenge lay not in the operation itself, but in the recipient's response to the transplanted organ. During the 1950s, Peter Medawar established that graft rejection was an active immunological response to genetically different tissue, providing the biological explanation for why transplantation beyond identical twins remained so difficult (5). His work shifted the central problem of transplantation from replacing the organ to controlling the recipient's response to it.

During the early 1960s and throughout the 1970s, Murray and other transplant pioneers, including Thomas Starzl and Roy Calne, translated this emerging understanding into increasingly effective clinical practice. Immunosuppressive regimens based on azathioprine and corticosteroids, followed by the introduction of Cyclosporin A, demonstrated that the immune response could be sufficiently suppressed to allow genetically non-identical grafts to survive and substantially improved transplant outcomes (6, 7). Murray later reflected that “the full story of successful organ transplantation in man weaves together three separate pathways: the study of renal disease, skin grafting in twins, and surgical determination” (1).

Transplantation thus revealed a broader principle of modern surgery: technical excellence alone could not determine the outcome of an operation. Surgical success increasingly depended on understanding the biological environment in which an intervention was performed and developing effective strategies to control it.

## Success meant responsibility

The ability to transplant organs successfully raised a different set of questions: who could be asked to donate, what risks were acceptable, and who should receive an organ that was hard to come by?

The ethical tension was already present during the first successful transplant. Ronald Herrick underwent major surgery while healthy, solely to benefit his brother (1, 2). Murray later described this as an unprecedented ethical dilemma: “The only remaining problem was the ethical decision concerning the removal of a healthy organ from a normal person for the benefit of someone else” (1).

As transplantation expanded beyond identical twins and living relatives, these ethical challenges became increasingly complex. Living donation required careful consideration of donor autonomy and acceptable risk, while the development of deceased donation came with even more fundamental ethical questions. Organs needed to be recovered while they remained viable, yet doing so required a clear determination that the donor had died. In 1968, the Harvard Medical School *ad hoc* Committee proposed criteria for irreversible coma as a new criterion, establishing the concept of death by neurologic criteria and addressing the ethical dilemma created by transplantation and modern life-sustaining technology (8).

At the same time, the scarcity of transplantable organs meant that even technically successful transplantation could not be offered to everyone who might benefit. Allocation therefore became an integral part of transplant medicine, requiring decisions not only about whether transplantation could succeed, but about who should have access to an organ, under what conditions and according to which principles.

The establishment of the United Network for Organ Sharing (UNOS) in 1986 marked an important step forward. As the organization responsible for operating the national Organ Procurement and Transplantation Network (OPTN), UNOS helped coordinate organ allocation across the United States and introduced a common framework for evaluating and improving transplant practice (9).

Transplantation thus expanded the boundaries of surgical responsibility beyond the patient and the surgery itself, making the ethical framework in which surgery was performed part of its success.

## Success meant teamwork

With the complexities of transplantation, success could not be achieved individually but required a collaborative approach. The outcome increasingly depended on the expertise of an entire team caring for the patient before, during, and after transplantation.

From the earliest transplant programs, surgeons worked alongside nephrologists, anesthesiologists, pathologists, nurses, and other specialists whose contributions were essential to patient selection, perioperative management, immunosuppression, and the recognition and treatment of complications (1). The collaboration between Joseph Murray and nephrologist John Merrill during the early development of kidney transplantation exemplified this emerging model: Murray's surgical skills were complemented by Merrill's knowledge of renal failure and dialysis, allowing transplantation to become part of a broader therapeutic strategy rather than an isolated operation (10). Murray himself later reflected that “if gold medals and prizes were awarded to institutions instead of individuals, the Peter Bent Brigham Hospital of 30 years ago would have qualified” (1).

As transplantation expanded to other organs, this collaborative model became an integral part of surgical care. The operation remained at the center of treatment, but its success was no longer the achievement of an individual surgeon but increasingly depended on coordinated decisions and care across multiple disciplines.

## Success measured long-term

As the field of transplantation matured, it became clear that discharging patients from their hospital stay marked only the beginning of the treatment. Graft function required carefully mastered monitoring with rejections and complications occurring months or even years later. Immunosuppression, still broad and unspecific to the day, came with many complications, and both graft and patient survival ultimately shaped the success of the intervention. Transplantation therefore demanded a long-term approach to measure outcomes, extending the endpoint from hospital discharge to years and, ultimately, decades of follow-up.

Joseph Murray was among the early transplant surgeons to put this principle into practice. He systematically documented graft function, patient survival, and causes of graft failure in his recipients, helping establish long-term outcome assessment as part of transplant care. In 1963, Murray brought together early transplant investigators from centers around the world to compile the growing experience with renal transplantation. By the following year, data on 342 renal transplants performed worldwide had been assembled (11). This early effort demonstrated the value of systematically collecting transplant outcomes and paved the way for longitudinal assessment on a much larger scale. The U.S. National Organ Transplant Act of 1984 provided the legal framework for national transplant outcome monitoring, followed by the establishment of the Scientific Registry of Transplant Recipients (SRTR) in 1987 to track outcomes across the transplant continuum, from waiting-list candidates and donors to recipients and long-term survival (12).

What began in transplantation as a need to optimize graft and patient outcomes became a broader principle of surgical care. Across surgical disciplines, success came to be defined not only by technical safety, but by the benefit it achieved over time.

## Success assessed by quality of life

Over years of follow-up, it became increasingly clear that a functioning graft did not necessarily mean a patient had returned to a normal life. Patients could survive for decades with a functioning organ while facing lifelong treatment-related complications, persistent symptoms, or limitations in their physical and overall well-being. As these challenges became recognized, conventional measures of graft and patient survival proved insufficient to capture the full outcome of transplantation.

Patient-reported outcome measures (PROMs) emerged to address this gap, bringing the patient's own experience into the assessment of transplant success. Rather than relying solely on graft function, survival, or clinician-assessed complications, PROMs capture dimensions going beyond standard clinical symptoms, including physical activity, treatment burden, and quality of life reported by the patient (13). This growing emphasis on the patient's perspective also strengthened the principles of patient-centered care and shared decision-making. Treatment goals increasingly incorporated not only what could be achieved medically, but also what patients valued themselves, including their priorities, preferences, and willingness to accept the burdens of lifelong treatment (14). Success was therefore understood in relation to the patient's life, rather than defined solely by clinical endpoints. This broader view of success reflects Joseph Murray's career arc as a reconstructive surgeon. His early work with skin grafts sparked his pioneering success in organ transplantation, which eventually brought him full circle back to his original passion for plastic surgery (15).

The evolution of transplantation thus came full circle: success was no longer defined simply by whether the operation worked or how long the graft lasted, but by what that success meant for the patient’s life.

## Conclusion

The trajectory of transplantation reflects a much broader transformation in surgery. For much of its history, surgery was defined by what the surgeon could accomplish: a successful operation performed, a complication avoided, a life saved. As surgical care became safer and more sophisticated, the definition of success expanded beyond the technical act itself—to understanding the biology that shaped its outcome, the team that made it possible, the durability of its effects, and ultimately the quality of life experienced by the patient (Figure 2).

**Figure 2:**
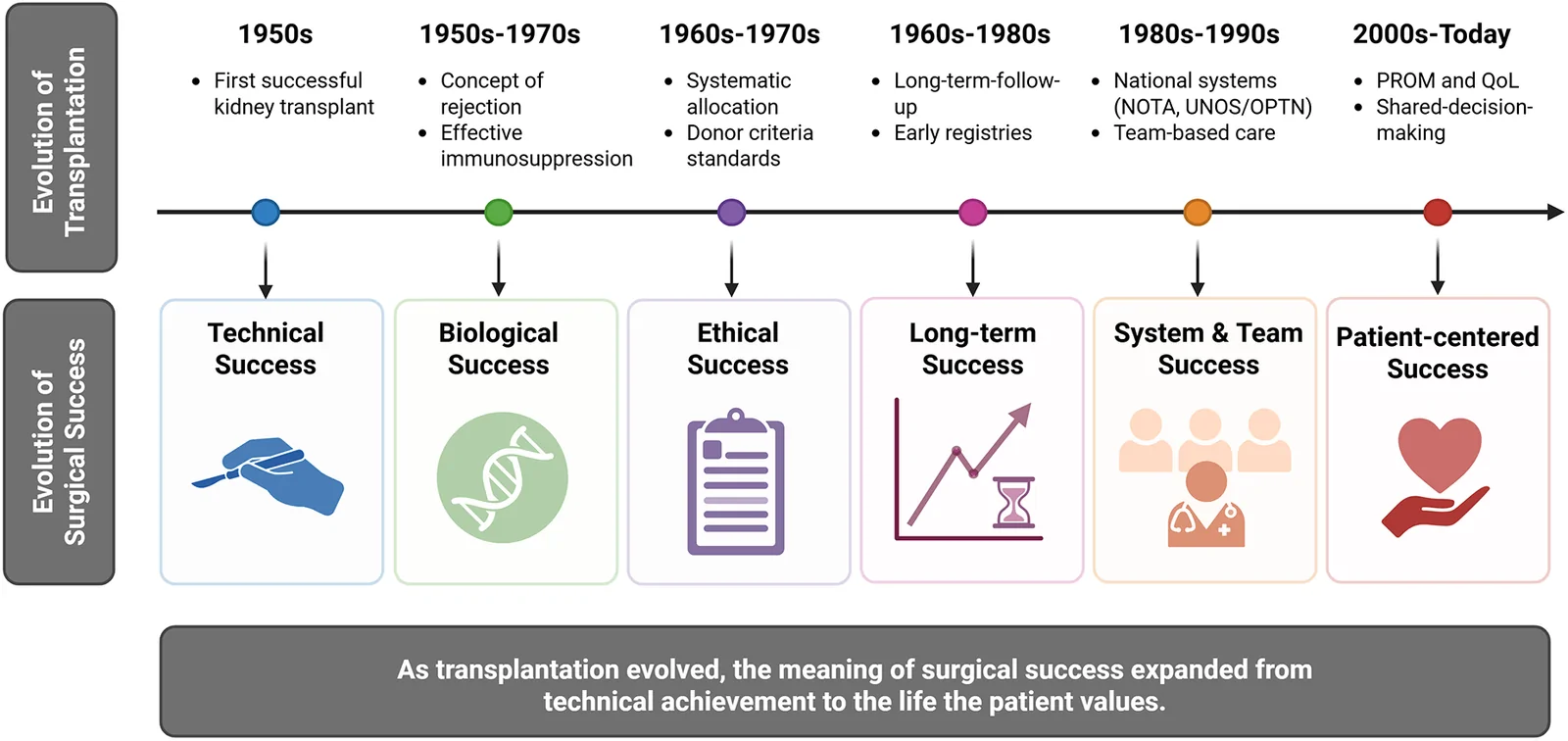
Evolution of transplantation and the changing definition of surgical success. As transplantation evolved, the meaning of surgical success expanded from technical achievement to the life the patient values.

Dr. Joseph Murray's legacy extends thus far beyond his pioneering work in organ transplantation, initiating a paradigm shift within surgical discourse, that prioritizes functional rehabilitation while focusing on long-term patient health and quality of life in a multi-disciplinary approach.

Modern surgical success is therefore no longer defined by what happens during an operation, but by what that operation ultimately makes possible.
